# Supplementary material for: Role and mechanism of NCAPD3 in promoting malignant behaviors in gastric cancer
Source: Front Pharmacol. 2024 Apr 22;15:1341039. doi: 10.3389/fphar.2024.1341039 (PMC11070777; doi:10.3389/fphar.2024.1341039)
Supplement: Supplementary file 11 [file DataSheet2.ZIP › GSEA/Oncogenic signature/my_analysis.Gsea.1599461962066/STK33_UP.html]

Details for gene set STK33\_UP[GSEA]

|  || Dataset | filtered\_dataset.sample\_info.cls#WT\_versus\_NCAPD3\_MUT |
| Phenotype | sample\_info.cls#WT\_versus\_NCAPD3\_MUT |
| Upregulated in class | NCAPD3\_MUT |
| GeneSet | STK33\_UP |
| Enrichment Score (ES) | -0.31311595 |
| Normalized Enrichment Score (NES) | -1.9541003 |
| Nominal p-value | 0.0 |
| FDR q-value | 0.028676562 |
| FWER p-Value | 0.173 |
Table: GSEA Results Summary

  

Fig 1: Enrichment plot: STK33\_UP      
 Profile of the Running ES Score & Positions of GeneSet Members on the Rank Ordered List

  

| SYMBOL | TITLE | RANK IN GENE LIST | RANK METRIC SCORE | RUNNING ES | CORE ENRICHMENT || 1 | 51351 | ZNF117 | 14 | 1.052 | 0.0251 | No |
| 2 | 23174 | ZCCHC14 | 27 | 1.004 | 0.0501 | No |
| 3 | 23253 | ANKRD12 | 34 | 0.977 | 0.0786 | No |
| 4 | 55727 | BTBD7 | 38 | 0.954 | 0.1085 | No |
| 5 | 2921 | CXCL3 | 57 | 0.884 | 0.1250 | No |
| 6 | 10161 | LPAR6 | 163 | 0.711 | 0.0720 | No |
| 7 | 145389 | SLC38A6 | 202 | 0.673 | 0.0668 | No |
| 8 | 400410 | ST20 | 235 | 0.647 | 0.0651 | No |
| 9 | 161582 | DYX1C1 | 253 | 0.626 | 0.0737 | No |
| 10 | 4238 | MFAP3 | 279 | 0.609 | 0.0758 | No |
| 11 | 84542 | KIAA1841 | 382 | 0.547 | 0.0195 | No |
| 12 | 2919 | CXCL1 | 405 | 0.534 | 0.0213 | No |
| 13 | 2920 | CXCL2 | 454 | 0.501 | 0.0030 | No |
| 14 | 10773 | ZBTB6 | 562 | 0.441 | -0.0606 | No |
| 15 | 84159 | ARID5B | 699 | 0.379 | -0.1476 | No |
| 16 | 220213 | OTUD1 | 889 | -0.304 | -0.2759 | No |
| 17 | 54532 | USP53 | 898 | -0.322 | -0.2710 | No |
| 18 | 301 | ANXA1 | 914 | -0.342 | -0.2704 | No |
| 19 | 4973 | OLR1 | 944 | -0.366 | -0.2794 | No |
| 20 | 3176 | HNMT | 991 | -0.398 | -0.2997 | Yes |
| 21 | 9586 | CREB5 | 1009 | -0.409 | -0.2984 | Yes |
| 22 | 2673 | GFPT1 | 1016 | -0.414 | -0.2889 | Yes |
| 23 | 57552 | NCEH1 | 1020 | -0.419 | -0.2770 | Yes |
| 24 | 23764 | MAFF | 1039 | -0.436 | -0.2755 | Yes |
| 25 | 80315 | CPEB4 | 1053 | -0.448 | -0.2700 | Yes |
| 26 | 6016 | RIT1 | 1072 | -0.458 | -0.2678 | Yes |
| 27 | 196394 | AMN1 | 1079 | -0.461 | -0.2566 | Yes |
| 28 | 9076 | CLDN1 | 1084 | -0.463 | -0.2440 | Yes |
| 29 | 7378 | UPP1 | 1111 | -0.484 | -0.2468 | Yes |
| 30 | 2012 | EMP1 | 1119 | -0.494 | -0.2353 | Yes |
| 31 | 604 | BCL6 | 1136 | -0.504 | -0.2300 | Yes |
| 32 | 6310 | ATXN1 | 1177 | -0.547 | -0.2409 | Yes |
| 33 | 153222 | CREBRF | 1187 | -0.559 | -0.2287 | Yes |
| 34 | 23657 | SLC7A11 | 1205 | -0.581 | -0.2216 | Yes |
| 35 | 10221 | TRIB1 | 1229 | -0.604 | -0.2182 | Yes |
| 36 | 22822 | PHLDA1 | 1255 | -0.643 | -0.2149 | Yes |
| 37 | 135114 | HINT3 | 1257 | -0.645 | -0.1939 | Yes |
| 38 | 11057 | ABHD2 | 1258 | -0.646 | -0.1721 | Yes |
| 39 | 57561 | ARRDC3 | 1324 | -0.744 | -0.1947 | Yes |
| 40 | 1839 | HBEGF | 1328 | -0.751 | -0.1717 | Yes |
| 41 | 5997 | RGS2 | 1336 | -0.770 | -0.1509 | Yes |
| 42 | 57568 | SIPA1L2 | 1347 | -0.788 | -0.1317 | Yes |
| 43 | 9966 | TNFSF15 | 1376 | -0.900 | -0.1220 | Yes |
| 44 | 9990 | SLC12A6 | 1379 | -0.922 | -0.0924 | Yes |
| 45 | 8867 | SYNJ1 | 1387 | -0.979 | -0.0646 | Yes |
| 46 | 26959 | HBP1 | 1395 | -1.073 | -0.0336 | Yes |
| 47 | 4312 | MMP1 | 1407 | -1.304 | 0.0022 | Yes |
Table: GSEA details [plain text format]

  

Fig 2: STK33\_UP      
 Blue-Pink O' Gram in the Space of the Analyzed GeneSet

  

Fig 3: STK33\_UP: Random ES distribution      
 Gene set null distribution of ES for **STK33\_UP**
